# Supplementary material for: Strengthening midwifery in the South-East Asian region: A scoping review of midwifery-related research
Source: PLoS One. 2023 Dec 15;18(12):e0294294. doi: 10.1371/journal.pone.0294294 (PMC10723687; doi:10.1371/journal.pone.0294294)
Supplement: S1 Table — (DOCX) [file pone.0294294.s001.docx]

**Supplementary Table 1. Search strategy**

| **Database** | **Search terms used** |
| --- | --- |
| **CENTRAL** | (midwi* OR nurse-midwi* OR “nurse midwi*” OR midwifery/ OR nurse midwives/)  AND ("southeast asia" OR "south east asia" OR "south-east asia" OR "south asia" OR Bangladesh* OR Nepal* OR Bhutan* OR Korea* OR India* OR Indonesia* OR Maldives OR Myanmar OR Burm* OR "Sri Lanka*" OR Thai* OR Timor* OR Democratic People’s Republic of Korea/ OR Timor-Leste/ OR Thailand/ OR Sri Lanka/ OR Myanmar/ OR Maldives/ OR Indonesia/ OR India/ OR Bhutan/ OR Nepal/ OR Bangladesh/ OR Asia, Southeastern/)  Limited to publication date between 2012-2022 |
| **CINAHL** | (midwi* OR nurse-midwi* OR “nurse midwi*” OR Midwifery/ OR Nurse Midwifery/ OR Maternal Health Services/)  AND (“Southeast Asia” OR “South East Asia” OR “South-east Asia” OR “South Asia” OR Bangladesh* OR Nepal* OR Bhutan* OR Korea* OR India* OR Indonesia* OR Maldives OR Myanmar OR Burm* OR “Sri Lanka*” OR Thai* OR Timor* OR Asia, Southeastern/ OR Bangladesh/ OR Nepal/ OR Bhutan/ OR North Korea/ OR India/ or Indonesia/ OR Indian Ocean Islands/ OR Myanmar/ OR Sri Lanka/ OR Thailand/ OR East Timor/)  Limited to publication date between 2012-2022 |
| **Dimensions** | Midwifery AND Southeast Asia  Limited to publication date between 2012 – 2022  Limited to Fields of Research: 4204 Midwifery or 4205 Nursing or 4203 Health Services and Systems |
| **Embase** | (Midwi* OR nurse-midwi* OR “nurse midwi*” OR Midwife/ OR Nurse Midwife/ OR Maternal health service/)  AND (“Southeast Asia” OR “South East Asia” OR South-east Asia” OR “South Asia” OR Bangladesh* OR Nepal* OR Bhutan* OR Korea* OR India* OR Indonesia* OR Maldives OR Myanmar OR Burm* OR “Sri Lanka*” OR Thai* OR Timor* OR Southeast Asia/ OR Southeastern Asia/ OR South Asia/ OR Bangladesh/ OR Nepal/ OR Bhutan/ OR North Korea/ OR India/ or Indonesia/ OR Maldives/ OR Myanmar/ OR Sri Lanka/ OR Thailand/ OR Timor-Leste/)  Limited to publication date between 2012-2022 |
| **Emcare** | (midwi* OR nurse-midwi* OR “nurse midwi*” OR Midwife/ OR Nurse midwife/ OR Maternal health service/)  AND ("South East Asia*" OR "Southeast Asia*" OR "South-East Asia*" OR "South Asia*" OR India OR Indonesia* OR Timor* OR Thai* OR Myanmar OR Burm* OR Nepal* OR Bhutan* OR Maldives OR "Sri Lanka*" OR Korea* OR Bangladesh* OR Southeast Asia/ OR South Asia/ OR Bangladesh/ OR Nepal/ OR Bhutan/ OR North Korea/ OR India/ OR Indonesia/ OR Maldives/ OR Myanmar/ OR Sri Lanka/ OR Thailand/ OR Timor-Leste/)  Limited to publication date between 2012-2022 |
| **Global Health** | (midwi* OR nurse-midwi* OR “nurse midwi*” OR Midwives/ OR Maternity Services)  AND ("South East Asia*" OR "Southeast Asia*" OR "South-East Asia*" OR "South Asia*" OR India* OR Indonesia* OR Timor* OR Thai* OR Myanmar OR Burm* OR Nepal* OR Bhutan* OR Maldives OR "Sri Lanka*" OR Korea* OR Bangladesh* OR South East Asia/ OR South Asia/ OR Bangladesh/ OR Nepal/ OR Bhutan/ OR Korea Democratic People’s Republic/ OR India/ OR Indonesia/ OR Maldives/ OR Myanmar/ OR Sri Lanka/ OR Thailand/ OR East Timor/)  Limited to publication date between 2012-2022 |
| **Google Scholar** | Midwifery AND South East Asia  Limited publication date between 2012-2022 |
| **Maternity & Infant Care** | ((midwi* OR nurse-midwi* OR "nurse midwi*" OR maternity)  AND ("South East Asia*" OR "Southeast Asia*" OR "South-East Asia*" OR "South Asia*" OR Bangladesh* OR Bhutan* OR Nepal* OR "Sri Lanka*" OR Maldives OR Myanmar OR Burm* or Thai* OR Indonesia* OR Timor* OR "North Korea*" OR India*)).mp. [mp=abstract, heading word, title]  Limited to publication date between 2012-2022 |
| **Medline** | (midwi* OR “nurse midwi*” OR “nurse-midwi*” OR Midwifery/ OR Nurse Midwives/ OR Maternal Health Services/)  AND (“Southeast Asia” OR “South East Asia” OR “South-east Asia” OR “South Asia” OR Bangladesh* OR Nepal* OR Bhutan* OR Korea* OR India* OR Indonesia* OR Maldives OR Myanmar OR Burm* OR “Sri Lanka*” OR Thai* OR Timor* OR Asia, Southeastern/ OR Bangladesh/ OR Bhutan/ OR Democratic People’s Republic of Korea/ OR India/ OR Indonesia/ OR Maldives/ OR Myanmar/ OR Nepal/ OR Sri Lanka/ OR Thailand/ OR Timor-Leste/)  Limited to publication date between 2012-2022 |
| **ProQuest** | ((MESH.EXACT("Maternal Health Services") OR MESH.EXACT("Nurse Midwives") OR MESH.EXACT("Midwifery")) OR noft(midwi* OR nurse-midwi* OR "nurse midwi*"))  AND (MESH.EXACT("Bhutan") OR MESH.EXACT("Indonesia") OR MESH.EXACT("Timor-Leste") OR MESH.EXACT("Sri Lanka") OR MESH.EXACT("Asia, Southeastern") OR MESH.EXACT("Democratic People's Republic of Korea") OR MESH.EXACT("Myanmar") OR MESH.EXACT("Nepal") OR MESH.EXACT("Bangladesh") OR MESH.EXACT("India") OR MESH.EXACT("Indian Ocean Islands") OR MESH.EXACT("Thailand"))  Limited to publication date between 2012-2022 |
| **PsychInfo** | (midwi* OR nurse-midwi* OR “nurse midwi*” OR Midwifery/)  AND ("Southeast Asia*" OR "South East Asia*" OR "South-East Asia*" OR “South Asia*” OR India or Bangladesh* OR Indonesia* OR Bhutan* OR Nepal* OR "Sri Lanka*" OR Maldives OR Timor* OR Korea* OR Myanmar OR Burm* OR Thai* OR Southeast Asian Cultural Groups/ OR South Asian Cultural Groups/)  Limited to publication date between 2012-2022 |
| **Scopus** | TITLE-ABS-KEY ((midwi* OR nurse-midwi* OR “nurse midwi*”)  AND (“Southeast Asia*” OR “South East Asia*” OR “South Asia*” OR Bangladesh* OR Bhutan* OR Nepal* OR India* OR “Sri Lanka*” OR Maldives OR Timor* OR Indonesia* OR Thai* OR Myanmar OR Burm* OR “North Korea*”))  AND PUBYEAR > 2011 AND PUBYEAR < 2023 |
| **Web of Science** | (midwi* OR nurse-midwi* OR "nurse midwi*")  AND ("South East Asia*" OR "Southeast Asia*" OR "South-East Asia*" OR "South Asia*" OR Bangladesh* OR Bhutan* OR Nepal* OR "Sri Lanka*" OR Maldives OR Myanmar OR Burm* OR Thai* OR Indonesia* OR Timor* OR "North Korea*" OR India*)  Limited to publication date between 2012-2022 |
