# Supplementary material for: Strengthening midwifery in the South-East Asian region: A scoping review of midwifery-related research
Source: PLoS One. 2023 Dec 15;18(12):e0294294. doi: 10.1371/journal.pone.0294294 (PMC10723687; doi:10.1371/journal.pone.0294294)
Supplement: S2 Table — (DOCX) [file pone.0294294.s002.docx]

**Supplementary Table 2. All included sources**

| **Source** | **Country** | **Study design** | **Setting** | **Midwives/Midwifery in focus** | **Focus area** | **Publication type** |
| --- | --- | --- | --- | --- | --- | --- |
| Acharya 2020 [1] | Nepal | Not reported | University hospitals | Midwifery faculties | CPD/post service education | Conference abstract |
| Acharya 2015 [2] | Nepal | Quantitative | Primary level health care institutions  Sub-health posts | Upgraded auxiliary nurse midwives  Auxiliary nurse midwives | CPD/post-service education | Journal article |
| Adnani 2018 [3] | Indonesia | Qualitative | Midwifery schools | Graduate midwives  Midwives  Midwifery educators | Pre-service education  Governance and regulation | Conference abstract |
| Adnani 2022 [4] | Indonesia | Qualitative | National | Midwifery education | Pre-service education  Governance and regulation | Journal article |
| Adnani 2022 [5] | Indonesia | Qualitative | Midwifery schools | Midwifery students  Graduate midwives  Midwives  Midwifery lecturers | Pre-service education | Journal article |
| Adnani 2022 [6] | Indonesia | Qualitative | Midwifery schools | Midwifery students  Graduate midwives  Midwives  Midwifery educators | Pre-service education  Governance and regulation | Journal article |
| Agrawal 2016 [7] | India | Quantitative | Public general nursing midwifery schools | Nursing and midwifery students | Pre-service education | Journal article |
| Agrawal 2021 [8] | India | Quantitative | Public sector nursing midwifery institutions | Midwifery students | Pre-service education | Journal article |
| Ahmed 2014 [9] | India | Quantitative | Tertiary care hospital | Midwives | CPD/post-service education | Conference abstract |
| Alam 2018 [10] | Bangladesh | Quantitative | Not reported | Midwives | CPD/post-service education | Journal article |
| Amalia 2021 [11] | Indonesia | Quantitative | Community health centre | Midwives | Workforce management | Journal article |
| Amp 2016 [12] | Indonesia | Quantitative | Government health centres | Midwives | CPD/post-service education | Journal article |
| Anawo Ameh 2015 [13] | Bangladesh | Quantitative | Not reported | Midwives | CPD/post-service education | Conference abstract |
| Andersen 2016 [14] | Nepal | Mixed methods | Primary public facilities | Auxiliary nurse midwives | CPD/post-service education  Practice or service delivery | Journal article |
| Anderson 2023* [15] | Bangladesh | Quantitative | Tertiary level teaching hospitals | Midwives | Practice or service delivery | Journal article |
| Anderson 2022 [16] | Bangladesh | Mixed methods | Government sub-district hospitals | Midwives  Stakeholders | Practice or service delivery  Leadership | Journal article |
| Anita 2018 [17] | Indonesia | Quantitative | Maternity hospitals  Independent midwifery practices | Midwives | Practice or service delivery | Journal article |
| Arifin 2018 [18] | Indonesia | Quantitative | Public health centres | Village midwives  Midwife coordinators | Leadership  Workforce management | Journal article |
| Arifin 2018 [19] | Indonesia | Quantitative | Public health centres | Village midwives | Practice or service delivery  Workforce management | Journal article |
| Arifin 2018 [20] | Indonesia | Quantitative | Public health centres | Village midwives  Midwife coordinators | Leadership  Workforce management | Journal article |
| Astutii 2020 [21] | Indonesia | Qualitative | Remote villages | Village midwives | Practice or service delivery  Workforce management | Journal article |
| Balasubramaniam 2018 [22] | India | Quantitative | Auxiliary nurse midwife training centres | Auxiliary nurse midwife students | Pre-service education | Journal article |
| Bardosono 2018 [23] | Indonesia | Quantitative | Not reported | Midwives | CPD/post-service education | Journal article |
| Barnabee 2014 [24] | Timor Leste | Mixed methods | Not reported | Midwives | Practice or service delivery  Workforce management | Conference abstract |
| Barua 2020 [25] | Bangladesh | Quantitative | Sub-district hospitals | Midwives | Practice or service delivery | Conference abstract |
| Beckingham 2022 [26] | India | Qualitative study | Specialist tertiary teaching hospital | Trainee midwives  Professional midwives | Pre-service education  Practice or service delivery | Journal article |
| Biswas 2018 [27] | Bangladesh | Qualitative | Teagarden health facilities | Professional midwives | Practice or service delivery | Journal article |
| Bogren 2012 [28] | Bangladesh  India  Nepal  Bhutan | Quantitative | International workshop | Midwifery education | Pre-service education  Governance and regulation | Journal article |
| Bogren 2013 [29] | Nepal | Mixed methods | National | Midwifery profession | Pre-service education  Governance and regulation  Workforce management | Journal article |
| Bogren 2015 [30] | Bangladesh | Qualitative | National | Midwifery profession  Midwifery leaders/stakeholders | Pre-service education  Governance and regulation  Workforce management | Journal article |
| Bogren 2016 [31] | Nepal | Qualitative | National | Midwifery profession  Midwifery leaders/stakeholders | Pre-service education  Governance and regulation  Workforce management | Journal article |
| Bogren 2017 [32] | Bangladesh | Qualitative | Education institutes | Midwifery faculty members | Pre-service education  Leadership  Governance and regulation | Journal article |
| Bogren 2018 [33] | Bangladesh | Quantitative | Public institutes/colleges | Midwifery education | Pre-service education | Journal article |
| Bogren 2018 [34] | Bangladesh  Nepal | Qualitative | International | Midwifery profession | Pre-service education  Governance and regulation | Journal article |
| Bogren 2018 [35] | Bangladesh | Qualitative | Public nursing institutes/colleges | Midwifery students | Pre-service education | Journal article |
| Bogren 2019 [36] | Bangladesh | Qualitative | Institutions and colleges | Midwifery educators | Pre-service education | Journal article |
| Bogren 2020 [37] | Bangladesh | Qualitative | Midwifery education institutions | Midwifery education | Pre-service education | Journal article |
| Boonduaylan 2022 [38] | Thailand | Quantitative | Labour and delivery units  Regional tertiary hospitals | Nurse-midwives | Workforce management | Journal article |
| Bull 2020 [39] | Indonesia | Qualitative | Public health centres  Private midwife-led clinics | Clinical facilitators | Pre-service education | Journal article |
| Byrskog 2019 [40] | Bangladesh | Qualitative | Public nursing institutes and colleges | Midwifery educators  Midwifery students | Pre-service education  Practice or service delivery | Journal article |
| Chiranthika Vithana 2015 [41] | Sri Lanka | Quantitative | Well woman clinics | Public health midwives | CPD/post-service education | Journal article |
| Damayanti 2019 [42] | Indonesia | Qualitative | Professional association  Health department  Independent midwifery practices | Independent practice midwives | Governance and regulation | Journal article |
| Das 2022 [43] | India | Quantitative | National | Auxiliary nurse midwives  Midwives | Pre-service education  Workforce management  Governance and regulation | Journal article |
| Das 2022 [44] | India | Qualitative | Community health centres | Auxiliary nurse midwives | Practice or service delivery  Leadership  Workforce management | Journal article |
| Dyer 2016 [45] | India | Qualitative | Primary health clinics  District hospitals | Nurse midwife mentors | CPD/post-service education  Leadership | Conference abstract |
| Ekawati 2019 [46] | Indonesia | Qualitative | Primary care | Midwives | Practice or service delivery | Conference abstract |
| Elison 2015 [47] | India  Thailand  Myanmar  Indonesia  Nepal | Qualitative | International | Midwifery profession | Governance and regulation | Journal article |
| Erlandsson 2018 [48] | Bangladesh | Mixed methods | Education institutes/colleges | Midwifery faculty staff members | Pre-service education  Leadership | Journal article |
| Erlandsson 2019 [49] | Bangladesh | Mixed methods | Education institutes | Midwifery educators | Pre-service education | Journal article |
| Fahriany 2018 [50] | Indonesia | Qualitative | Education institute | Midwifery students | Pre-service education | Journal article |
| Faucher 2016 [51] | India | Quantitative | Hospital | Nurse-midwives | CPD/post-service education | Journal article |
| Febrianty 2020 [52] | Indonesia | Quantitative | Neonatal health services | Midwives | Workforce management | Journal article |
| Ferina 2019 [53] | Indonesia | Quantitative | Public health centre | Midwives | CPD/post-service education  Practice or service delivery | Journal article |
| Fiore-Silvast 2013 [54] | India | Mixed methods | Clinics  Home visits | Nurse midwives | Practice or service delivery | Journal article |
| Fitriana 2022 [55] | Indonesia | Qualitative | Healthcare settings | Midwives | Practice or service delivery | Journal article |
| Foss 2020 [56] | India | Mixed methods | District and private hospitals  Primary health clinics  Sub-stations  Schools  Villages | Auxiliary nurse midwives | CPD/post-service education | Journal article |
| Fullerton 2016 [57] | Myanmar | Quantitative | Midwifery schools | Midwifery education programs | Pre-service education | Journal article |
| Giri 2012 [58] | India | Quantitative | Nursing school | Auxiliary nurse and midwife [revised] students | Pre-service education  Workforce management | Journal article |
| Gusti 2018 [59] | Indonesia | Quantitative | Community health centres | Midwives | Practice or service delivery | Journal article |
| Halimsetiono 2018 [60] | Indonesia | Quantitative | Primary healthcare centres | Midwives | Workforce management | Journal article |
| Hardjanti 2017 [61] | Indonesia | Quantitative | Hospital | Midwives | Workforce management | Journal article |
| Harris 2021 [62] | Bangladesh | Quantitative | Refugee camp and host community | Midwives | CPD/post-service education  Practice or service delivery | Meeting abstract |
| Hasritawati 2021 [63] | Indonesia | Quantitative | Public health centre | Midwives | CPD/post-service education | Journal article |
| Herlina 2021 [64] | Indonesia | Mixed methods | Independent practices of midwifery | Independent practice midwives  Hospital midwives | Practice or service delivery | Journal article |
| Hermasari 2019 [65] | Indonesia | Qualitative | University faculty of medicine | Undergraduate midwifery students | Pre-service education | Journal article |
| Hettiarachchi 2017 [66] | Sri Lanka | Quantitative | Workshop-based | Public health midwives | CPD/post-service education | Conference abstract |
| Hikmandayani 2021 [67] | Indonesia | Quantitative | Ministry of health polytechnic | Midwifery students | Pre-service education | Journal article |
| Hou 2016 [68] | Timor Leste | Quantitative | National | Midwives | Workforce management | Journal article |
| Husaini 2017 [69] | Indonesia | Quantitative | Midwifery academy | Midwifery academic students | Pre-service education | Journal article |
| Ilankoon 2022 [70] | Sri Lanka | Quantitative | Community-based setting | Public health midwives | CPD/post-service education | Journal article |
| Indrayani 2017 [71] | Indonesia | Qualitative | Villages | Village midwives | Workforce management  Practice or service delivery | Journal article |
| Indrayani 2017 [72] | Indonesia | Qualitative | Not reported | Midwives | Pre-service education | Journal article |
| Indrayani 2023* [73] | Indonesia | Quantitative | Primary health centres  Independent midwifery clinics | Midwives | CPD/post-service education | Journal article |
| Indriani 2020 [74] | Indonesia | Qualitative | Primary healthcare centres  Hospitals | Midwives | Practice or service delivery | Journal article |
| Infanti 2015 [75] | Sri Lanka | Qualitative | Health clinics | Public health midwives | Practice or service delivery | Journal article |
| Irawan 2016 [76] | Indonesia | Qualitative | University | Midwives  Graduate midwives | Practice or service delivery | Conference article |
| Jan 2014 [77] | India | Quantitative | Not reported | Midwife trainers | CPD/post-service education | Conference abstract |
| Janjua 2017 [78] | India | Qualitative | Primary health centres | Nurse-midwife mentors  Auxiliary nurse midwives | CPD/post-service education  Leadership | Conference abstract |
| Januraheni 2019 [79] | Indonesia | Quantitative | Health centres  Hospitals | Midwives | Practice or service delivery | Journal article |
| Jayanti 2017 [80] | Indonesia | Quantitative | Community health centres | Midwives | Workforce management  Leadership | Journal article |
| Jayathilake 2016 [81] | Sri Lanka | Qualitative | Tertiary care hospitals’ intrapartum and postpartum units | Midwifery trained registered nurses | Practice or service delivery | Journal article |
| Jayatilleke 2015 [82] | Sri Lanka | Quantitative | Primary health care services | Public health midwives | CPD/post-service education | Journal article |
| Johariyah 2020 [83] | Indonesia | Qualitative | Public health centres | Midwives | Practice or service delivery  Workforce management | Journal article |
| Kardinah 2014 [84] | Indonesia | Quantitative | Not reported | Midwives | CPD/post-service education  Practice or service delivery | Journal article |
| Karvande 2020 [85] | India | Mixed methods | Sub-centres  Primary health centres | Auxiliary nurse midwives | CPD/post-service education | Journal article |
| Karvande 2018 [86] | India | Qualitative | Community health centres  Primary health centre  Health subcentres | Auxiliary nurse midwives | Pre-service education  CPD/post-service education | Journal article |
| Khatun 2020 [87] | Bangladesh | Quantitative | Upazila health complexes | Midwives | Practice or service delivery | Journal article |
| Khatun 2021 [88] | Bangladesh | Qualitative | Clinical placement sites | Midwifery students  Graduate midwives | Pre service education  CPD/post-service education | Journal article |
| Kodali 2021 [89] | India | Mixed methods | Primary health centres | Auxiliary nurse midwives | Practice or service delivery | Journal article |
| Korake 2019 [90] | India | Qualitative | Not reported | Auxiliary nurse midwives | Workforce management | Journal article |
| Korake 2019 [91] | India | Qualitative | Sub-centres | Auxiliary nurse midwives | Workforce management | Journal article |
| Kostania 2016 [92] | Indonesia | Quantitative | Health polytechnic | Midwifery students | Pre-service education | Conference article |
| Kozuki 2016 [93] | Nepal | Quantitative study | Home visits | Auxiliary nurse midwives | CPD/post-service education  Practice or service delivery | Abstract |
| Kumar 2013 [94] | India | Qualitative | Health sub-centre | Auxiliary nurse midwives | Workforce management | Journal article |
| Kundaryanti 2018 [95] | Indonesia | Quantitative | Public health centre | Midwives | Practice or service delivery | Journal article |
| Kurniawan 2019 [96] | Indonesia | Not reported | Health centres | Village midwives | Practice or service delivery | Journal article |
| Lai 2022 [97] | Indonesia | Quantitative | Not reported | Midwives | Practice or service delivery | Journal article |
| Lestari 2017 [98] | Indonesia | Qualitative | University | Undergraduate midwifery students | Pre-service education | Journal article |
| Lestari 2018 [99] | Indonesia | Mixed methods | Educational institutions | Healthcare faculty members | Pre-service education | Journal article |
| Lubis 2019 [100] | Indonesia | Qualitative | Private midwifery clinics | Private midwives | Practice or service delivery | Journal article |
| Lubis 2022 [101] | Indonesia | Quantitative | Health centres | Midwives | Practice or service delivery | Journal article |
| Mahato 2018 [102] | Nepal | Qualitative study | Birthing centres | Auxiliary nurse midwives | CPD/post-service education | Journal article |
| Marfu’ah 2016 [103] | Indonesia | Quantitative | Antenatal care services | Midwives | Workforce management | Journal article |
| Markam 2018 [104] | Indonesia | Mixed methods study | Primary healthcare centres | Midwives | Practice or service delivery | Journal article |
| Martina 2021 [105] | Indonesia | Quantitative study | University | Midwifery students | Pre-service education | Journal article |
| Mayra 2021 [106] | India | Qualitative study | National | Midwifery and nursing leadership stakeholders | Pre-service education  Leadership  Workforce management  Governance and regulation | Journal article |
| Meilani 2019 [107] | Indonesia | Quantitative study | Mother and children clinics | Midwives | CPD/post-service education  Practice or service delivery | Journal article |
| Mirna 2020 [108] | Indonesia | Quantitative study | University | Midwifery lecturers | CPD/post-service delivery | Journal article |
| Molina 2020 [109] | India | Quantitative study | Primary and community health centres | Auxiliary nurse midwives | CPD/post-service education  Practice or service delivery | Journal article |
| Morrison 2015 [110] | Nepal | Mixed methods | Sub-health posts  Health posts  Primary health centres  Hospitals | Auxiliary nurse midwives | Workforce management | Journal article |
| Mousumi 2018 [111] | Bangladesh | Quantitative study | Sub-district hospitals | Midwives | CPD/post-service education  Practice or service delivery | Conference abstract |
| Mousumi 2018 [112] | Bangladesh | Mixed methods study | Sub-district hospitals | Midwives | CPD/post-service education  Practice or service delivery | Journal article |
| Ngana 2012 [113] | Indonesia | Mixed methods | Rural clinics | Village midwives | Practice or service delivery | Journal article |
| Nikmah 2021 [114] | Indonesia | Qualitative | National | Midwifery profession | Pre-service education  Governance and regulation | Journal article |
| Nirmala 2017 [115] | Indonesia | Quantitative | University | Midwifery students | Pre-service education | Journal article |
| Noya 2022 [116] | Myanmar | Qualitative | Midwifery training school | Midwifery students | Pre-service education | Journal article |
| Nugraheny 2016 [117] | Indonesia | Mixed methods | School of midwifery | Student midwives  Midwifery supervisors | Pre-service education | Journal article |
| Nurfatimah 2021 [118] | Indonesia | Quantitative | Health centre | Midwives | Practice or service delivery | Journal article |
| Olson 2012 [119] | Indonesia | Quantitative | Community-based settings | Community midwives | Practice or service delivery | Journal article |
| Olson 2015 [120] | Indonesia | Quantitative | Midwives’ places of work | Community-based midwives | CPD/post-service education | Journal article |
| Palfreyman 2019 [121] | Sri Lanka | Qualitative | Hospital antenatal clinics | Public health midwives | Practice or service delivery | Journal article |
| Pankaj 2012 [122] | India | Qualitative | Not reported | Auxiliary nurse midwives | CPD/post-service education | Journal article |
| Panthong 2014 [123] | Thailand | Mixed methods | Community hospital  Sub-district promoting hospital | Nurse-midwives | CPD/post-service education  Practice or service delivery | Journal article |
| Panuntun 2019 [124] | Indonesia | Qualitative | Not reported | Village midwives | Practice or service delivery  Workforce management | Journal article |
| Parveen 2021 [125] | Bangladesh | Qualitative | Teaching institutes | Midwifery educators  Clinical midwives | Pre-service education | Journal article |
| Patel 2023* [126] | India | Quantitative | Primary, secondary, and tertiary level care facilities | Midwives | CPD/post-service education | Journal article |
| Pathiraja 2016 [127] | Sri Lanka | Quantitative | Community based | Public health midwives | Workforce management | Conference abstract |
| Pemo 2019 [128] | Bhutan | Qualitative | Hospital reproductive health unit | Midwives | Practice or service delivery | Journal article |
| Permatasari 2017 [129] | Indonesia | Quantitative | Community health centres | Village midwives | Workforce management  Leadership | Journal article |
| Pramono 2022 [130] | Indonesia | Qualitative | Maternity care facility | Midwives | Practice or service delivery | Journal article |
| Prasetyo 2020 [131] | Indonesia | Quantitative | University | Midwifery students | Pre-service education | Journal article |
| Prastyoningsih 2022 [132] | Indonesia | Qualitative study | Government-owned midwifery institution | Midwives | CPD/post-service education | Journal article |
| Pratinidhi 2013 [133] | India | Mixed methods | Primary health centres  Hospitals | Nurse midwives  Auxiliary nurse midwives | Practice or service delivery | Journal article |
| Pratiwi 2021 [134] | Indonesia | Quantitative study | Health centres | Midwives | CPD/post-service education | Journal article |
| Pratiwi 2021 [135] | Indonesia | Quantitative study | Health centres | Midwives | CPD/post-service education | Journal article |
| Puri 2014 [136] | Nepal | Quantitative study | Primary health care centre; health posts and sub-health posts; village development committees | Auxiliary nurse midwives | CPD/post-service education  Practice or service delivery | Journal article |
| Purnami 2019 [137] | Indonesia | Quantitative | Primary healthcare | Midwives | Workforce management | Journal article |
| Purnima 2013 [138] | India | Quantitative | Outreach centres | Auxiliary nurse midwives | Practice or service delivery | Conference abstract |
| Purohit 2017 [139] | India | Quantitative | Government health centres | Auxiliary nurse midwives | Workforce management | Journal article |
| Pyone 2019 [140] | India | Qualitative | Health facilities | Auxiliary nurse midwives | Workforce management  Governance and regulation | Journal article |
| Rahmadani 2019 [141] | Indonesia | Quantitative | Community health centres | Public health centre midwives | Workforce management | Journal article |
| Rahman 2018 [142] | Bangladesh | Mixed methods | Nursing colleges and institutes | Midwifery education | Pre-service education | Conference abstract |
| Rahman 2018 [143] | Bangladesh | Not reported | Not reported | Midwives | Pre-service education  Workforce management | Conference abstract |
| Rahman 2018 [144] | Indonesia | Qualitative | Health office | Midwives | Practice or service delivery | Journal article |
| Rajvanshi 2022 [145] | India | Quantitative | Sub-centres  Villages | Auxiliary nurse midwives | Practice or service delivery | Journal article |
| Ransing 2020 [146] | India | Quantitative | Tertiary care hospital antenatal clinic | Auxiliary nurse midwives | Practice or service delivery | Journal article |
| Ray 2019 [147] | India | Quantitative | Community development blocks | Auxiliary nurse midwives | CPD/post-service delivery | Journal article |
| Ria 2020 [148] | Indonesia | Quantitative | University | Midwifery students | Pre-service education | Journal article |
| Risnah 2021 [149] | Indonesia | Quantitative | Primary health centres | Midwives | CPD/post-service education | Journal article |
| Rodrigo 2012 [150] | Sri Lanka | Mixed methods | Remote medical officer of health area | Public health midwives | Practice or service delivery | Journal article |
| Romo 2016 [151] | India | Quantitative | Primary health clinics | Nurse midwife mentors | CPD/post-service education | Conference abstract |
| Rosa 2020 [152] | Indonesia | Quantitative | Not reported | Midwives | Practice or service delivery | Journal article |
| Rosmaria 2019 [153] | Indonesia | Mixed methods | Hospitals  Public health centres  Independent midwifery practices  Village maternity posts  Midwifery clinics  Auxiliary health centre | Midwives | CPD/post-service education  Practice or service delivery | Journal article |
| Rukmini 2013 [154] | Indonesia | Mixed methods | Health centres | Midwives | Practice or service delivery  Governance and regulation | Journal article |
| Samidah 2021 [155] | Indonesia | Mixed methods | Educational institutions | Student midwives | Pre-service education | Journal article |
| Saragih 2019 [156] | Indonesia | Quantitative | University | Midwifery students | Pre-service education | Journal article |
| Sardillah 2020 [157] | Indonesia | Quantitative | Midwifery education institution | Midwifery students | Pre-service education | Journal article |
| Sari 2022 [158] | Indonesia | Quantitative | Public health centres | Midwives | Practice or service delivery | Journal article |
| Sethi 2019 [159] | Indonesia | Quantitative | Public and private health facilities | Midwives | CPD/post-service education | Journal article |
| Sharma 2013 [160] | India | Qualitative | Tertiary hospital  District hospital  Community health centres | Staff nurses with midwifery registration | Workforce management  Governance and regulation | Journal article |
| Sharma 2015 [161] | India | Quantitative | Educational institutions | Midwifery students | Pre-service education | Journal article |
| Sharma 2018 [162] | India | Quantitative | Educational institutions | Midwifery students | Pre-service education | Journal article |
| Stellata 2022 [163] | Indonesia | Qualitative | Public health centres | Village midwives | Practice or service delivery | Journal article |
| Sugino 2014 [164] | Indonesia | Qualitative | Public health centres | Midwives | CPD/post-service education  Workforce management | Journal article |
| Sujana 2019 [165] | Indonesia | Mixed methods | Midwifery department of health polytechnic | Midwifery students | Pre-service education | Journal article |
| Sumiaty 2021 [166] | Indonesia | Quantitative | Professional midwives’ association | Midwives | Practice or service delivery | Journal article |
| Surianto 2018 [167] | Indonesia | Qualitative | Not reported | Independent midwives | Governance and regulation | Journal article |
| Susanti 2022 [168] | Indonesia | Mixed methods | Not reported | Midwives | Practice or service delivery | Journal article |
| Susianti 2017 [169] | Indonesia | Quantitative | Community health centres | Midwives | Practice or service delivery | Journal article |
| Thacker 2013 [170] | India | Quantitative | Primary health centres | Auxiliary nurse midwives | Practice or service delivery | Journal article |
| Than 2017 [171] | Myanmar | Qualitative | Township hospital  Community gathering places  NGO office | Auxiliary midwives | Workforce management | Journal article |
| Than 2017 [172] | Myanmar | Quantitative | Rural health centres  Maternal and child health centre | Auxiliary midwives | CPD/post-service education  Practice or service delivery | Journal article |
| Than 2018 [173] | Myanmar | Mixed methods | Community and township health systems | Auxiliary midwives | Practice or service delivery  Workforce management | Journal article |
| Than 2019 [174] | Myanmar | Qualitative | Community and township health systems | Auxiliary midwives | Practice or service delivery  Workforce management | Journal article |
| Titaley 2017 [175] | Indonesia | Qualitative | Village based | Village midwives | Practice or service delivery | Journal article |
| Ulfa 2021 [176] | Indonesia | Quantitative | Midwifery department of schools | Midwifery students | Pre-service education | Journal article |
| Ulfa 2021 [177] | Indonesia | Quantitative | Health polytechnic school department of midwifery | Midwifery students | Pre-service education | Journal article |
| Van Der Wal 2016 [178] | Myanmar | Qualitative | Non-government organisation | Auxiliary midwives | Practice or service delivery  Workforce management | Conference abstract |
| Vedam 2022 [179] | India | Qualitative study | Not reported | Nurse practitioners in midwifery | Pre-service education  Leadership  Governance and regulation  Workforce management | Journal article |
| Vohra 2022 [180] | India | Mixed method | Primary health centres  Subcentres | Auxiliary nurse midwives | Practice or service delivery | Journal article |
| Wahidah 2018 [181] | Indonesia | Quantitative | Community health centre | Midwives | Workforce management | Journal article |
| Wahyuni 2017 [182] | Indonesia | Quantitative | Hospitals | Midwives | CPD/post-service education  Governance and regulation | Journal article |
| Wangmo 2016 [183] | Myanmar | Quantitative | Village-based | Auxiliary midwives | Workforce management | Journal article |
| Warriner 2015 [184] | Nepal | Quantitative | Not reported | Midwives | Practice or service delivery | Conference abstract |
| Weaver 2013 [185] | Indonesia | Quantitative | National | Village midwives | Practice or service delivery | Journal article |
| Widyawati 2015 [186] | Indonesia | Qualitative | Public health centres | Nurse-midwives | Practice or service delivery | Journal article |
| Wild 2019 [187] | Timor Leste | Qualitative | Health posts  Community health centres  Hospitals  Domestic violence referral services | Midwives | Practice or service delivery | Journal article |
| Wrammer 2017 [188] | Nepal | Qualitative | Tertiary level government hospital | Nurse midwives | CPD/in-service education | Journal article |
| Wyatt 2022 [189] | Sri Lanka | Qualitative | Antenatal clinics | Public health midwives | Practice or service delivery | Journal article |
| Yadav 2022 [190] | India | Mixed methods | Hospitals | Independent nurse midwifery practitioners | Workforce management | Journal article |
| Yanti 2015 [191] | Indonesia | Quantitative | Schools of midwifery | Midwifery students | Pre-service education | Journal article |
| Youngwanichsetha 2020 [192] | Thailand | Qualitative | University midwifery course | Graduate nursing students enrolled in midwifery course | Pre-service education | Journal article |
| Zaman 2020 [193] | Bangladesh | Mixed methods | Government or NGO health facilities Public or private education institutes | Midwives  Midwifery students | Pre-service education  CPD/post-service education  Workforce management | Journal article |
| Zin 2015 [194] | Myanmar | Mixed methods | Project schools | Midwifery education | Pre-service education  Governance and regulation | Conference abstract |
| Zullianti 2022 [195] | Indonesia | Qualitative | Health centre | Midwives | Post-service education  Practice or service delivery | Journal article |

** These sources were officially published in 2023; however, they were captured in our search strategy as they were available online in 2022*

**References**

1. Acharya A, Shrestha U, Aradhya R, Fernando P, Kalaunee A. Implementation of Simulation Based Trainings among Midwifery Faculties in Nepal. BMJ Simul Technol Enhanc Learn. 2020;6(Suppl 1):A34-A35.doi: 10.1136/bmjstel-2020-aspihconf.58.

2. Acharya D, Paudel R. Assessment of critical knowledge on maternal and newborn care services among primary level nurse mid-wives in Kapilvastu District of Nepal. Kathmandu Univ Med J. 2015;52(4):351-6.

3. Adnani QES, McAra-Couper J, Gilkison A. Partnerships to strengthen midwifery education: findings from a qualitative study in Indonesia. Women Birth. 2018;31(S1):S17-S8. doi: 10.1016/j.wombi.2018.08.058.

4. Adnani QES, Gilkison A, McAra-Couper J. A historical narrative of the development of midwifery education in Indonesia. Women Birth. 2022;36(1):e175-1781.doi: 10.1016/j.wombi.2022.06.007.

5. Adnani QES, Gilkison A, McAra-Couper J. Strengthening midwifery education through clinical experience: Findings from a qualitative study in Indonesia. Women Birth. 2022;35(1):87-95. doi: 10.1016/j.wombi.2021.03.002.

6. Adnani QES, Gilkison A, McAra-Couper J. The interplay of structural and external factors for strengthening midwifery education in Indonesia. Sex Reprod Healthc. 2022;33(2022):100734. doi: 10.1016/j.srhc.2022.100734.

7. Agrawal N, Kumar S, Balasubramaniam SM, Bhargava S, Sinha P, Bakshi B, Sood B. Effectiveness of virtual classroom training in improving the knowledge and key maternal neonatal health skills of general nurse midwifery students in Bihar, India: A pre- and post-intervention study. Nurse Educ Today. 2016;36(2016):293-7. doi: 10.1016/j.nedt.2015.07.022.

8. Agrawal N, Bhargava S, Usmanova G, Srivastava A, Kumar S, Mahajan S, Gautam A, Shah H, Sinha P, Kumar R, Bairagi A, Kumar S, Sood B. Evaluating the effect of strengthening nurse midwifery pre-service education in two Indian states: A single group pre - and post - intervention study. Nurse Educ Today. 2021;96(2021):104640. doi: 10.1016/j.nedt.2020.104640.

9. Ahmed A, Singha B, Lahkar M. Knowledge, attitude and practice of pharmacovigilance among the nurses and midwives in a tertiary care hospital. Indian J Pharmacol. 2014;46:S53.

10. Alam R, Anderson R, Johnson J, Karim A. Strengthening midwife-led services through mentorship at sub-district hospitals in Bangladesh. Int J Gynaecol Obstet. 2018;143:286-7.doi: 10.1002/ijgo.12582.

11. Amalia VR, Prasetya H, Murti B. Path Analysis on Factors Affecting Job Performance of Midwives at Community Health Centers in Mojokerto, East Java. J Health Policy Manage. 2021;6(1):21-34. doi: 10.26911/thejhpm.2021.06.01.03.

12. Amp S, Lumbanraja SN. Exploring midwives’ knowledge about myelomeningocele in North Western Indonesia. Turkish Online Journal of Educational Technology. 2016(November Special Issue):997-1001.

13. Anawo Ameh C, on behalf of CMNH LSTM. Increasing the capacity of healthcare workers to provide emergency obstetric and early newborn care (EMONC) in Sub-Saharan Africa and South Asia. Int J Gynaecol Obstet. 2015;131(Suppl 5):E36.

14. Andersen KL, Basnett I, Shrestha DR, Shrestha MK, Shah M, Aryal S. Expansion of Safe Abortion Services in Nepal Through Auxiliary Nurse-Midwife Provision of Medical Abortion, 2011-2013. J Midwifery Womens Health. 2016;61:177-84. doi:10.1111/jmwh.12419.

15. Anderson R, Williams A, Emdadul Hoque DM, Jess N, Shahjahan F, Hossain A, Bogren M. Implementing midwifery services in public tertiary medical college hospitals in Bangladesh: A longitudinal study. Women Birth. 2023;36(3):299-304. doi: 10.1016/j.wombi.2022.09.006.

16. Anderson R, Williams A, Jess N, Read JM, Limmer M. The impact of professional midwives and mentoring on the quality and availability of maternity care in government sub-district hospitals in Bangladesh: a mixed-methods observational study. BMC Pregnancy Childbirth. 2022;22(1):827. doi: 10.1186/s12884-022-05096-x.

17. Anita W. The implementation of documentation by midwives in Pekanbaru. Kesmas: National Public Health Journal. 2018;13(1):43-7. doi: 10:21109/kesmas.v13i1.1403.

18. Arifin S, Suhariadi F, Damayanti NA. The influence of leadership style and domicile to power distance of midwife coordinators and village midwives in Hulu Sungai Tengah District. Indian J Public Health Res Dev. 2018;9(12):635-8. doi: 10.5958/0976-5506.2018.01908.3.

19. Arifin S, Suhariadi F, Damayanti NA. The role of domicile on the achievement of village midwife performances in antenatal care through a job involvement. Indian J Public Health Res Dev. 2018;9(1):258-62.

20. Arifin S, Suhariadi F, Damayanti NA. The influence of leadership style of midwife coordinator toward the performance of village midwives on antenatal care through the job involvement. Indian J Public Health Res Dev. 2018;9(1):249-52.

21. Astutii H, Indrayani, Harianis S. Challenges for midwives in remote areas: An ethnography study. Pak J Med Health Sci. 2021;14(4):1691-7.

22. Balasubramaniam SM, Bhargava S, Agrawal N, Asif R, Chawngthu L, Sinha P, Kumar S, Sood B. Blending virtual with conventional learning to improve student midwifery skills in India. Nurse Educ Pract. 2018;28(2018):163-7. doi: 10.1016/j.nepr.2017.10.028.

23. Bardosono S, Hildayani R, Chandra DN, Basrowi RW, Wibowo Y. The knowledge retention after continuing health education among midwives in Indonesia. Med J Indones. 2018;27:128-33. doi: 10.13181/mji.v27i2.2413.

24. Barnabee G, Harrison M, Mercer MA, O'Malley G. Midwives' perceptions of an innovative mHealth technology's impact on their work and job satisfaction. Ann Glob Health. 2014;80(3).

25. Barua HR, Dewan G, Ziko MAH. Professional midwife led maternal and newborn health care in primary health facilities of Rangamati district of Bangladesh. J Obstet Gynaecol Res. 2020;46:92. doi: 10.1111/jog.14455

26. Beckingham A, Downe S, Fernandez E, Reed B, Kaur I, Aziz N, Kingdon C. Implementing Professional Midwife-Led Maternity Care in India for Healthy Pregnant Women: A Community Case Study. Front Public Health. 2022;10:875595. doi:10.3389/fpubh.2022.875595

27. Biswas A, Anderson R, Doraiswamy S, Abdullah ASM, Purno N, Rahman F, Halim A. Timely referral saves the lives of mothers and newborns: Midwifery led continuum of care in marginalized teagarden communities – A qualitative case study in Bangladesh. F1000research. 2018;7:365. doi: 10.12688/f1000research.13605.1

28. Bogren MU, Wiseman A, Berg M. Midwifery education, regulation and association in six South Asian countries - A descriptive report. Sex Reprod Healthc. 2012;3(2012):67-72. doi: 10.1016/j.srhc.2012.03.004

29. Bogren MU, van Teijlingen E, Berg M. Where midwives are not yet recognised: a feasibility study of professional midwives in Nepal. Midwifery. 2013;29(10):1103-9. doi: 10.1016/j.midw.2013.07.019.

30. Bogren MU, Wigert H, Edgren L, Berg M. Towards a midwifery profession in Bangladesh--a systems approach for a complex world. BMC Pregnancy Childbirth. 2015;15(325). doi: 10.1186/s12884-015-0740-8.

31. Bogren MU, Berg M, Edgren L, van Teijlingen E, Wigert H. Shaping the midwifery profession in Nepal - Uncovering actors' connections using a Complex Adaptive Systems framework. Sex Reprod Healthc. 2016;10:48-55.

32. Bogren M, Doraiswamy S, Erlandsson K. Building a new generation of midwifery faculty members in Bangladesh. J Asian Midwives. 2017;4(2):52-8.

33. Bogren M, Doraiswamy S, Erlandsson K. Development of a context specific accreditation assessment tool for affirming quality midwifery education in Bangladesh. Midwifery. 2018;61:74-80. doi: 10.1016/j.midw.2018.02.021.

34. Bogren M, Erlandsson K. Opportunities, challenges and strategies when building a midwifery profession. Findings from a qualitative study in Bangladesh and Nepal. Sex Reprod Healthc. 2018;16:45-9. doi: 10.1016/j.srhc.2018.02.003.

35. Bogren M, Erlandsson K, Members of the Midwifery Faculty Master’s degree holders in Sexual and Reproductive Health Rights, Byrskog U. What prevents midwifery quality care in Bangladesh? A focus group enquiry with midwifery students. BMC Health Serv Res. 2018;18(639). doi: 10.1186/s12913-018-3447-5.

36. Bogren M, Rosengren J, Erlandsson K, Berg M. Build professional competence and Equip with strategies to empower midwifery students – An interview study evaluating a simulation-based learning course for midwifery educators in Bangladesh. Nurse Educ Pract. 2019;35:27-31. doi: 10.1016/j.nepr.2019.01.002.

37. Bogren M, Banu A, Parvin S, Chowdhury M, Erlandsson, K. Implementation of a context-specific accreditation assessment tool for affirming quality midwifery education in Bangladesh: a qualitative research study. Glob Health Action. 2020;13(1): 1761642. doi: 10.1080/16549716.2020.1761642

38. Boonduaylan S, Deoisres, Wacharasin, C. A causal model of job stress among Thai nurse-midwives. Front Nurs. 2022;9(2):215-23. doi: 10.2478/fon-2022-0016.

39. Bull A, Geraghty S, Dube M, Budiani NN, Mahayati NMD, Utarini GAE, Sriasih NGK, Arini KN, Adnyani S, Noviani NW. Facilitating midwifery students on an international placement: Clinical facilitator perspectives. Nurse Educ Pract. 2020;48:102869. doi: 10.1016/j.nepr.2020.102869

40. Byrskog U, Akther HA, Khatoon Z, Bogren M, Erlandsson K. Social, economic and professional barriers influencing midwives' realities in Bangladesh: A qualitative study of midwifery educators preparing midwifery students for clinical reality. Evid Based Midwifery. 2019;17(1):19-26.

41. Chiranthika Vithana PVS, Ariyaratne, MAY, Jayawardana PL. Effectiveness of an educational intervention among public health midwives on breast cancer early detection in the district of Gampaha, Sri Lanka. Asian Pac J Cancer Prev. 2015;16(1):227-32. doi: 10.7314/APJCP.2015.16.1.227

42. Damayanti FN, Absori A, Wardiono K. Legal protection of midwives based on professional justice in midwifery practices. Indian J Public Health Res Dev. 2019;10(4):437-41. doi: 10.5958/0976-5506.2019.00734.4

43. Das MK, Singh D. Nurses and Midwives Human Resource for Health and Their Education in India: A Situational Analysis. Florence Nightingale Journal of Nursing. 2022;30(1):9-17. doi: 10.5152/FNJN.2021.21013.

44. Das P, Ramani S, Newton-Lewis T, Nagpal P, Khalil K, Gharai D, Das S, Kammowanee R. "We are nurses-what can we say?": power asymmetries and Auxiliary Nurse Midwives in an Indian state. Sex Reprod Health Matters. 2022;29(2):2031598. doi: 10.1080/26410397.2022.2031598.

45. Dyer J, Cohen S, Christmas A, Spencer P, Taylor J, Frank H, Sterne J, Walker D. Designing and implementing an in-situ emergency obstetric and neonatal care [EmONC] simulation and team-training curriculum for midwife mentors to drive quality improvement in Bihar, India. Ann Glob Health. 2016;82(3):353.

46. Ekawati FM, Licqurish S, Gunn J, Lau P. Management of hypertensive disorders of pregnancy (HDP) in Indonesian primary care settings: The views of stakeholders. Aust J Prim Health, 25, xiv. doi: 10.1071/PYv25n3abs.

47. Elison NK, Verani AR, McCarthy C. National nursing and midwifery legislation in countries of South-East Asia with high HIV burdens. WHO South East Asia Journal of Public Health. 2015;4(1):12-9.

48. Erlandsson K, Doraiswamy S, Wallin L, Bogren M. Capacity building of midwifery faculty to implement a 3-years midwifery diploma curriculum in Bangladesh: A process evaluation of a mentorship programme. Nurse Educ Pract. 2018;29(2018):212-8. doi: 10.1016/j.nepr.2018.02.006

49. Erlandsson K, Byrskog U, Osman F, Pedersen C, Hatakka M, Klingberg-Allvin M. Evaluating a model for the capacity building of midwifery educators in Bangladesh through a blended, web-based master's programme. Glob Health Action. 2019;12(1): 1652022. doi: 10.1080/16549716.2019.1652022

50. Fahriany F, Nuraeni N. Need analysis of English needs of midwifery students in Indonesia. In: Madya S, Hamied F, Renandya WA, Coombe C, Basthomi Y, editors. ELT in Asia in the Digital Era: Global Citizenship and Identity. London: Routledge; 2018. pp. 173-80.

51. Faucher MA, Riley C, Prater L, Reddy MP. Midwives in India: a delayed cord clamping intervention using simulation. Int Nurs Rev. 2016;63(3):437-44. doi: 10.1111/inr.12264.

52. Febrianty D, Husaini, Ilmi B, Panghiyangani R, Marlinae L. The influence factors of the performance of midwives on the neonatal health services in Balangan District. Indian J Public Health Res Dev. 2020;11(6):1222-6.

53. Ferina, Purwara BH, Setiawati EP, Susiarno H, Abdurrahman M, Sukandar H. Knowledge of midwives on IUD counseling. SEAJOM: Southeast Asian J Midwifery. 2019;5(1):33-8.

54. Fiore-Silvast B, Hartung C, Iyengar K, Iyengar S, Israel-Ballard K, Perin N, Anderson R. Mobile video for patient education: The midwives' perspective. Proceedings of the 3rd ACM Symposium on Computing for Development. 2013; Bangalore, India. doi: 10.1145/2442882.2442885

55. Fitriana F, Ningtyas WS, Dewi ER. Providing mental healthcare for postpartum women in Indonesia: a qualitative phenomenological study. Br J Midwifery 2022;30(12):692-9.

56. Foss M, le May A, Gobbi M. The development and testing of a co-designed distance learning programme to improve the knowledge of trained auxiliary nurse midwives in normal midwifery practice in central India. J Res Nurs. 2020;25(6-7):541-58. doi: 10.1177/1744987120952836

57. Fullerton JT, Johnson P, Lobe E, Myint KH, Aung NN, Moe T, Linn NA. A Rapid Assessment Tool for affirming good practice in midwifery education programming. Midwifery. 2016;34:36-41. doi: 10.1016/j.midw.2016.01.008

58. Giri R, Mukhopadhyay A, Mallik S, Sarkar S, Debnath A, Patra P. A study on self concept and adjustment of auxiliary nursing and midwifery [revised] students in a selected school of nursing, Purulia, West Bengal. J Indian Medical Assoc. 2012;110(7):485-7.

59. Gusti TE, Tamtomo D, Murti B. Determinants of midwife performance on antenatal care in Surakarta and Karanganyar, Central Java. J Health Policy Manag. 2018;3(1):11-9. doi: 10.26911/thejhpm.2018.03.01.02

60. Halimsetiono E, Rochmah TN, Suminar DR. Role stress, personality type, burnout, and performance of midwives towards postnatal care program achievement in Surabaya City. Kesmas: National Public Health Journal. 2018;13(2):81-6. doi: 10.21109/kesmas.v13i2.1223

61. Hardjanti IW, Noermijati, Dewanto A. Influence of quality of work life towards psychological well-being and turnover intention of nurses and midwives in hospital. Kesmas: National Public Health Journal. 2017;12(1):7-14. doi: 10.21109/kesmas.v12i1.1144

62. Harris L, Qureshi H, Ibgui T, Hawkins H, Harris D, Alkarim T. Comparison of the neonatal resuscitation education [Helping Babies Breathe] and resource needs between selected healthcare facilities in Bangladesh, Pakistan, and Yemen. Pediatrics. 2021;147(3):236-7. doi: 10.1542/peds.147.3MA3.236

63. Hasritawati, Madeni B. Relation of Normal Childbirth Care Training to Midwives Knowledge and Attitudes at Bebesen Health Center Bebesen District of Central Aceh Regency in 2021. Bioscientia Medicina: J Biomedicine Translational Res. 2021;5(11):1123-7. doi: 10.32539/bsm.v5i11.426

64. Herlina N, Fratidhina Y, Ambariani, Mulyati S2, Martini T. Development of a patient safety model in independent practice of midwifery in bogor regency, West Java, Indonesia. Indian J Forensic Med Toxicol. 2021;15(3):3843-9.

65. Hermasari BK, Rahayu GR, Claramita M. How does portfolio assess interprofessional learning among medical and midwifery students? Int J Eval Res Educ. 2019;8(3):392-400. doi: 10.11591/ijere.v8i3.20245

66. Hettiarachchi S, Kitnasamy G, Gopi D, Poobalan R, Mahendran R, Kumara P, Yogaraj Y, Shamra F, Bandara B, Gowritharan P. Effect of a low-cost multi-disciplinary team-led experiential workshop for midwives on dysphagia in children with cerebral palsy. Dev Med Child Neurol; 2017:80. doi: 10.1111/dmcn.13456

67. Hikmandayani, Ahmad M, Syarif S, Budu, Idris I. Stang. Learning media based on augmented reality [AR] increased the skill of physical examination of the integumentary system of pregnant women in midwifery students. Gac Sanit. 2021;35(S2):S302-S305. doi: 10.1016/j.gaceta.2021.10.040

68. Hou X, Witter S, Zaman RU, Engelhardt K, Hafidz F, Julia F, Lemiere C, Sullivan EB, Saldanha E, Palu T, Lievens T. What do health workers in Timor-Leste want, know and do? Findings from a national health labour market survey. Hum Resour Health. 2016;14(1):69. doi: 10.1186/s12960-016-0164-1

69. Husaini, Panghiyangani R, Saputra M. The effects of health education toward HIV/AIDS knowledge and attitude on banjarbaru midwife academy students 2016. Indian J Public Health Res Dev. 2017;8(2):332-6. doi: 10.5958/0976-5506.2017.00136.X

70. Ilankoon IMPS, Goonewardena CSE, Fernandopulle RC, Perera PPR. Public Health Midwives’ Perceptions, Challenges and Attitudes Towards Health Education on Vulvovaginal Discharge. Sri Lankan Journal of Nursing. 2022;1(1):11-20.

71. Indrayani FH, Sarbini AS, Andriyani A, Sari DN, Bachtar F, Bebasari M, Agus M, Mukarramah S, Suseno MR, Norfitri R, Marliana Y. The midwifery practice challenges in the rural populations of Indonesia. Res J Med Sci. 2017;11(1):40-5.

72. Indrayani, Husin F, Hilmanto D, Ritha A, Rumintang BI, Daswati, Maternity D, Budihartini EMS, Djami MEU, Kartikawati SL, Purwanti Y, Rahayu YP, Sefrina Y, Isnaini YS, Wirakusumah FF. Expectations and prospects regarding the midwife services in Indonesia. J South India Medicolegal Assoc. 2017;9(2):114-22. doi: 10.5281/zenodo.1322983.

73. Indrayani, Stacey T, Merchant HA, Darwin Z. Gravimetric assessment of postpartum blood loss: training and implementation in a low resource setting. Aust J Adv Nurs. 2023;40(1):21-9. doi: 10.37464/2023.401.693.

74. Indriani D, Damayanti NA, Teguh D, Ardian M, Suhargono H, Urbaya S, Wulandari RD, Nindya TS, Ernawaty E, Putri NK, Ridlo IA. The maternal referral mobile application system for minimizing the risk of childbirth. J Public Health Res. 2020;9:1813

75. Infanti JJ, Lund R, Muzrif MM, Schei B, Wijewardena K, on behalf of the ADVANCE study team. Addressing domestic violence through antenatal care in Sri Lanka's plantation estates: Contributions of public health midwives. Social Sci Med. 2015;145:35-43. doi: 10.1016/j.socscimed.2015.09.037.

76. Irawan YS, Soegijoko S, Koesoema AP, Utama DQ, Riyani A, Isdiningrat AA, Isdiningrat IS, Husin F. Towards sustainable mHealth applications for maternal and child health: The case of Sahabat Bundaku-an integrated mobile application for mothers and midwives. Proceedings of the IEEE Region 10 Conference (TENCON); 2016.

77. Jan H, Devarajan S, Nair V, Vishwanatha R, Basu H, Sarris I. Evaluating the feasibility, rating, and potential impact of CALMED, a low-tech simulation-based training for obstetric and neonatal emergencies. BJOG. 2014;121(S2):185. doi: 10.1111/1471-0528.12790.

78. Janjua AT, Dyer J, Spindler H, Sterne J, Cohen S, Morgan M, Christmas A, Bharat Shah M, Das A, Walker DD. Video analysis system as a tool to improve the quality of basic emergency obstetric and neonatal care through simulation training in Bihar, India. Ann Glob Health. 2017;83(1): 104.

79. Januraheni NLP, Gau M-L. Comparison between pregnant women' preferences and midwives' performance in prenatal congenital anomaly screenings counseling in Indonesia. SEAJOM: The Southeast Asia J Midwifery. 2017;3(2):40-51.doi: 10.36749/seajom.v3i2.13.

80. Jayanti ND, Tamtomo DG, Sulaeman, ES. Path analysis on the effects of motivation and other factors on midwives performance of preeclampsia management in Malang, East Java. J Health Policy Manag. 2017;2(2):137-46. doi: 10.26911/thejhpm.2017.02.02.05.

81. Jayathilake S, Jayasuriya-Illesinghe V, Perera R, Molligoda H, Samarasinghe K. 'Competent, but not allowed to blossom': Midwifery-trained registered nurses' perceptions of their service: A qualitative study in Sri Lanka. J Asian Midwives. 2016;3(2):39-54.

82. Jayatilleke AC, Yoshikawa K, Yasuoka J, Poudel KC, Fernando N, Jayatilleke AU, Jimba M. Training Sri Lankan public health midwives on intimate partner violence: a pre- and post-intervention study. BMC Public Health. 2015;15(1): 331. doi: 10.1186/s12889-015-1674-9.

83. Johariyah, Widyawati, Nurdiati DS. Exploring Indonesian Midwive's Experience of Preeclampsia Screening and Community Health Volunteer's Role to Prevent Maternal and Fetal Complications: A Qualitative Study at the Community Level. Sys Rev Pharm. 2020;11(11):186-93.

84. Kardinah D, Anderson BO, Duggan C, Ali IA, Thomas DB. Limited effectiveness of screening mammography in addition to clinical breast examination by trained nurse midwives in rural Jakarta, Indonesia. Int J Cancer. 2014;134(5):1250-5. doi: 10.1002/ijc.28442.

85. Karvande S, Purohit V, Gopalakrishnan SS, Subha Sri B, Mathai M, Mistry N. Building capacities of Auxiliary Nurse Midwives (ANMs) through a complementary mix of directed and self-directed skill-based learning—A case study in Pune District, Western India. Hum Resour Health. 2020;18(1):45. doi: 10.1186/s12960-020-00485-9.

86. Karvande S, Sonawane D, Samal J, Mistry N. Family planning training needs of auxiliary nurse midwives in Jharkhand, India: Lessons from an assessment. The Natl Med J India. 2018;3(2):73-8.

87. Khatun A, Jhumu MA, Gregson S, Kemp J. Developing and Piloting a Midwifery Audit Tool in Bangladesh’s Upazila Health Complexes (UHCs). J Asian Midwives. 2020;7(1):7-22.

88. Khatun M, Akter P, Yunus S, Alam K, Pedersen C, Byrskog U, Erlandsson K. Challenges to implement evidence-based midwifery care in Bangladesh. An interview study with medical doctors mentoring health care providers. Sex Reprod Healthc. 2022;31:100692. doi: 10.1016/j.srhc.2021.100692.

89. Kodali PB, Das S. Acceptance of mHealth technologies among Auxiliary Nurse Midwives in Andhra Pradesh, India: A mixed method study. Medical Science. 2021;25(111):1052-60.

90. Korake R, Bhore N. Assessment of societal experiences of Auxiliary Nurse Midwives working in Sub-center of Pune district, Maharashtra. Pravara Med Rev. 2019;11(1):8-11.

91. Korake RV, Bhore N, Bhamare S. A study to assess personal experiences of Auxiliary Nurse Midwives working in Sub-center of Pune district - exploratory study. Int J Healthc Biomed Res. 2019;7(3):20-7.

92. Kostania G. The differences between the influence of group investigation and Jigsaw cooperative learning methods toward students' learning outcomes viewed from midwifery students' scientific attitude. Proceedings of the 2nd International Conference on Public Health. 2016:8-18. doi: 10.17501/icoph.2016.2102.

93. Kozuki N, Mullany LC, Khatry SK, Ghimire RK, Paudel S, Blakemore K, Bird C, Tielsch JM, LeClerq SC, Katz J. Validity of home-based sonographic diagnosis of obstetric risk factors by auxiliary nurse midwives in rural Nepal. Lancet Glob Health. 2016;4.

94. Kumar A, Mahapatro M. The cutting edge in the blunt space: an anthropological construct of auxiliary nurse midwives' social world in the community. Healthc Low-Resource Settings. 2013;1:e10.

95. Kundaryanti R, Effendy N, Masrul, Asmawi. Quality of antenatal care according to perspective of patient and midwives in sukamulya public health centre, Tangerang City. Pak J Med Health Sci. 2018;12(4):1783-5.

96. Kurniawan R, Siregar KN, Martiana NS, Wardhani IK. mHealth development for village midwives to improve the performance of the maternal health program in the babakan madang sub-district, Bogor, Indonesia. Indian J Public Health Res Dev. 2019;10(7):981-6. doi: 10.5958/0976-5506.2019.01706.6.

97. Lai SL, Tey NP. Midwives as Drivers of Contraceptive Uptake: Evidence From Indonesia Demographic and Health Surveys. Asia Pac J Public Health. 2022;34(2-3):213-20. doi: 10.1177/10105395211058810

98. Lestari E, Yuliyanti S, Rosdiana I, Surani E, Luailiyah A. Contributing Factors of Acceptance and Rejection to Interprofessional Education: Undergraduate Students’ Perception. Online J Health Allied Sci. 2017;16(1):1-9.

99. Lestari E, Stalmeijer RE, Widyandana D, Scherpbier A. Understanding attitude of health care professional teachers toward interprofessional health care collaboration and education in a Southeast Asian country. J Multidiscip Healthc. 2018;11:557-71.

100. Lubis D, Wulandari LPL, Suariyani NLP, Adhi KT, Andajani S. Private Midwives' Perceptions of Barriers and Enabling Factors to Voluntary Counseling and HIV Testing in Bali, Indonesia. Kesmas: National Public Health Journal. 2019;14(1):14-20. doi: 10.21109/kesmas.v14i1.2708.

101. Lubis AUN, Lubis, DH, Harahap SW. Analysis of the Relationship of the Characteristics, Knowledge And Attitude of the Midwife With Completion of Partograph Filling in Normal Delivery Care. International Journal of Public Health Excellence. 2022;19(2):93-8. doi: 10.55299/ijphe.v1i2.39

102. Mahato PK, van Teijlingen E, Simkhada PP, Angell C, Ireland J, THET team. Qualitative evaluation of mental health training of auxiliary nurse midwives in rural Nepal. Nurse Educ Today. 2018;66:44-50. doi: 10.1016/j.nedt.2018.03.025.

103. Marfu'ah S, Tamtomo D, Suryono A. Effect of psychological factors and workload on midwife performance in the integrated antenatal care in Pati, Central Java. J Maternal Child Health. 2016;1(3):138-45. doi: 10.26911/thejmch.2016.01.03.01.

104. Markam H, Hochheiser H, Kuntoro K, Notobroto HB. Exploring Midwives' Need and Intention to Adopt Electronic Integrated Antenatal Care. Perspectives in Health Information Management. 2018;15.

105. Martina SE, Simanjuntak EH. The implementation of mini-cex on self-efficacy among midwifery student of sari mutiara Indonesia university. Eur J Mol Clin Med. 2021;8(2):2064-9.

106. Mayra K, Padmadas SS, Matthews Z. Challenges and needed reforms in midwifery and nursing regulatory systems in India: Implications for education and practice. PLoS ONE. 2021;16(5):e0251331. doi: 10.1371/journal.pone.0251331.

107. Meilani N, Setiyawati N, Barasa SO. Midwife's role in the mother-to-child transmission prevention program in primary health care in Yogyakarta. Kesmas: National Public Health Journal. 2019;14(2):88-94. doi: 10.21109/kesmas.v14i2.2774.

108. Mirna, Baso YS, Ramadhany S, Idris I, Bahar B, Jibril. The improvementof the role of lecturer in the learning process through e-learning models in associate degreeof midwifery, Megarezky University, Makassar. Eur J Mol Clin Med. 2020;7(7):4917-23.

109. Molina RL, Neal BJ, Bobanski L, Singh VP, Neville BA, Delaney MM, Lipsitz S, Karlage A, Shetye M, Semrau KEA. Nurses' and auxiliary nurse midwives' adherence to essential birth practices with peer coaching in Uttar Pradesh, India: a secondary analysis of the BetterBirth trial. Implementation Science. 2020;15(1):1. doi: 10.1186/s13012-019-0962-7.

110. Morrison J, Batura N, Thapa R, Basnyat R, Skordis-Worrall J. Validating a tool to measure auxiliary nurse midwife and nurse motivation in rural Nepal. Hum Resour Health. 2015;13(1):13-30. doi: 10.1186/s12960-015-0021-7.

111. Mousumi MNR, J.; Currie, S.; Anderson, R.; Doraiswamy, S.; Reinhardt, S.; Businge, L. Midwifery led maternity care [MLC] improves quality of labour and delivery care in Bangladesh. International Journal of Gynecology and Obstetrics. 2018;143:264-5.

112. Mousumi M, Rahman, JN, Currie S, Atieno I, Anderson R, Reinhardt S, Toscano M, Shahnaz P. Low dose-high frequency learning approaches improve the competency of midwives: Bangladesh experience. Int J Gyneacol Obstet. 2018;143:261-2. doi: 10.1002/ijgo.12582.

113. Ngana FR, Myers BA, Belton S. Health reporting system in two subdistricts in Eastern Indonesia: Highlighting the role of village midwives. Midwifery. 2012;28(6):809-15. doi: 10.1016/j.midw.2011.09.005.

114. Nikmah US, Wicaksono DDA. Quo Vadis Of Diversification Of Midwifery Education In Indonesia. Bul Penelit Sist Kesehat. 2021;24(2):119-27.

115. Nirmala SA, Judistiani TD. Does an Integrated Midwifery Curriculum Build a Better Understanding of Midwifery Profession is About Among First-Year Midwifery Student at Faculty of Medicine Padjadjaran University? Adv Sci Lett. 2018;24(12):9781-3. doi: 10.1166/asl.2018.13139

116. Noya A, Oguro M, Horiuchi S. Professional learning using storytelling videos of childbirth experiences: A qualitative pilot study of student midwives' perceptions in Myanmar. Jpn J Nurs Sci. 2022;19(2):e12456. doi: 10.1111/jjns.12456.

117. Nugraheny E, Claramita M, Rahayu GR, Kumara A. Feedback in the nonshifting context of the midwifery clinical education in Indonesia: A mixed methods study. Iranian Journal of Nursing and Midwifery Research. 2016;21(6):628-34. doi: 10.4103/1735-9066.197671.

118. Nurfatimah N, Ramadhan K, Entoh C, Longgupa LW, Hafid F. Continuity of Midwifery Care Implementation to Reduce Stunting. Open Access Maced J Medical Sci. 2021;9(E):1512-6. doi: 10.3889/oamjms.2021.7062.

119. Olson KR, Caldwell A, Sihombing M, Guarino AJ, Nelson BD. Community-based newborn resuscitation among frontline providers in a low-resource country. Int J Gynaecol Obstet. 2012;119(3):244-7. doi: 10.1016/j.ijgo.2012.07.012.

120. Olson KR, Caldwell A, Sihombing M, Guarino AJ, Nelson BD, Petersen R. Assessing self-efficacy of frontline providers to perform newborn resuscitation in a low-resource setting. Resuscitation. 2015;89:58-63. doi: 10.1016/j.resuscitation.2015.01.008

121. Palfreyman A. Helping or heightening vulnerability? Midwives as arbiters of risk for women experiencing self-directed violence in urban Sri Lanka. Qual Health Res. 2018;29(10):1383-94.

122. Prasad P, Arya RS, Bansal M, Singh SP. A comparative study to assess the impact of 6 days core competency training of the ANMs of Damoh District of Madhya. Natl J Community Med. 2012;3(1):121-4.

123. Panthong A, Parisunyakul S, Kantaruksa K, Sriareporn P, McDonald TTA. Development of a Midwifery Care Model for a Rural Community. Pac Rim Int J Nurs Res 2014;18(4):343-56.

124. Panuntun S, Karsidi R, Murti B, Akhyar M. The role of midwives and traditional birth attendant partnership program in empowering traditional birth attendant to improve maternal health in Klaten, Central Java. J Maternal Child Health. 2019;4(4):279-86. doi: 10.26911/thejmch.2019.04.04.07.

125. Parveen S, Akter N, Zahan M, Erlandsson K, Byrskog U. Addressing violence against women within the midwifery curriculum in Bangladesh. A focus group discussion inquiry. J Asian Midwives. 2021;8(1):15-34.

126. Patel AB, Bang A, Kurhe K, Bhargav S, Hibberd PL. What Helping Babies Breathe knowledge and skills are formidable for healthcare workers? Front Pediatr. 2023;10:891266. doi: 10.3389/fped.2022.891266.

127. Pathiraja I, Fonseka P, Mant D. Is Burnout among community midwives just a problem of high-income countries? Cross-sectional study from Sri Lanka. Inj Prev. 2016;22(Suppl 2):A70. doi: 10.1136/injuryprev-2016-042156.192

128. Pemo K, Phillips D, Hutchinson AM. Midwives’ perceptions of barriers to exclusive breastfeeding in Bhutan: A qualitative study. Women Birth. 2020;33:e377-e84. doi: 10.1016/j.wombi.2019.07.003.

129. Permatasari RD, Budihastuti, UR, Tamtomo D. Effect of socio-demographic factor and democratic leadership on village midwife performance in IUD contraceptive service in Jombang District, East Java. J Health Policy Manag. 2017;2(1):16-27. doi: 10.26911/thejhpm.2017.02.01.02.

130. Pramono A, Smith J, Bourke S, Desborough J. How midwives and nurses experience implementing ten steps to successful breastfeeding: a qualitative case study in an Indonesian maternity care facility. Int Breastfeed J. 2022;17:84. doi: 10.1186/s13006-022-00524-2.

131. Prasetyo B, Anis W, Amalia RB, Izzati D, Ningtyas WS, Jayanti RD, Fitriana F. The effect of repetition simulation after debrief method on learning satisfaction and learning achievement in midwifery students at Universitas Airlangga, Indonesia. Indian J Forensic Med Toxicol. 2020;14(4):4431-5.

132. Prastyoningsih A, Rohmah AN, Prastika DA, Kanita MW, Pratiwi AM, Umarianti T, Andikatyas YR. Continuing Midwifery Education: Midwifery Encouragements of Continuing Professional Education in Profession Stage in Indonesia: A Qualitative Study. Open Access Maced J Medical Sci. 2022;10(G):311-7. doi: 10.3889/oamjms.2022.8655.

133. Pratinidhi AK, Javadekar SS, Shrotri AN, Gandham SV, Patil A, Patil KS. Feasibility of use of color-coded rings by nurse midwives: an appropriate technology based on partographic principles. Indian J Community Med. 2013;38(3):157-61. doi: 10.4103/0970-0218.116352.

134. Pratiwi IG, Hamidiyanti BYF, Arifin A, Ristrini, Bachtiar A, Putro G. Effect of virtual reality for increasing midwives skills in intra uterine device training. Rawal Medical J. 2021;46(4):866-8.

135. Pratiwi IG, Hamidiyanti BYF, Arifin A, Husin F, Pandudita R, Ristrini R, Bachtiar A, Putro G, Dramawan A, Diarti MW. Virtual Reality Improves The Knowledge of Midwives in IUD (Intra Uterine Device) Training. Jurnal Kesehatan Prima. 2021;15(1):74-82.

136. Puri M, Tamang A, Shrestha P, Joshi D. The role of auxiliary nurse-midwives and community health volunteers in expanding access to medical abortion in rural Nepal. Reprod Health Matters. 2015;22(44):94-103. doi: 10.1016/S0968-8080(14)43784-4.

137. Purnami CT, Suwondo A, Sawitri DR, Sumarni S, Hadisaputro S, Lazuardi L. Psychometric measurement of perceived stress among midwives at primary health care province of central java indonesia. Indian J Public Health Res Dev. 2019;10(3):804-9. doi: 10.5958/0976-5506.2019.00600.4.

138. Purnima J, Divya P, Panda RM. Tobacco Control in Pregnant Women: Gaps and opportunities in practices of nurse midwives in outreach centres in India. Respir Med. 2013;107:S17. doi: 10.1016/S0954-6111(13)70062-3.

139. Purohit B, Vasava P. Role stress among auxiliary nurses midwives in Gujarat, India. BMC Health Serv Res. 2017;17:69. doi: 10.1186/s12913-017-2033-6.

140. Pyone T, Karvande S, Gopalakrishnan S, Purohit V, Nelson S, Balakrishnan SS, Mistry N, Mathai M. Factors governing the performance of Auxiliary Nurse Midwives in India: A study in Pune district. PLoS ONE. 2019;14(12):e0226831. doi: 10.1371/journal.pone.0226831.

141. Rahmadani LN, Budihastuti UR, Poncorini E. Does health center have contextual effect on midwife performance? multilevel analysis evidence from East Kalimantan, Indonesia. J Health Policy Manag. 2019;4(3):150-60. doi: 10.26911/thejhpm.2019.04.03.01.

142. Rahman JN, Mousumi M, Rozario A, Currie S. Are the midwifery schools prepared for midwives? An assessment of all midwifery schools in banglades. Int J Gyneacol Obstet. 2018;143:464. doi: 10.1002/ijgo.12582.

143. Rahman JN, Mousumi M, Currie S, Bogren M, Reinherd S, Businge L, Akhter S. Can paramedics (FWVS) be upgraded to ICM standard midwives? Assessment of FWVS in Bangladesh. Int J Gyneacol Obstet. 2018;143:226. doi: 10.1002/ijgo.12582.

144. Rahman F, Marlinae L, Setyaningrum R, Putri AO, Hilmiyati. The role of midwife through antenatal class pregnancy for improvement delivery assistance with professional health workers. Indian J Public Health Res Dev. 2018;9(1). doi: 10.5958/0976-5506.2018.00031.1.

145. Rajvanshi H, Islam F, Kashyap V, Pathak R, Agarwalla R, Gupta E, Lal AA. Assessment of frontline health workers in providing services for malaria elimination in the tribal district of Mandla, Madhya Pradesh. J Fam Med Prim Care. 2022;11(11):7233-62. doi: 10.4103/jfmpc.jfmpc_1005_22

146. Ransing R, Deshpande SN, Shete SR, Patil I, Kukreti P, Raghuveer P, Mahadevaiah M, Bhosale N, Ramesh VO, Puri M, Bantwal P. Assessing antenatal depression in primary care with the PHQ-2 and PHQ-9: Can It Be carried Out by auxiliary nurse midwife [ANM]? Asian J Psychiatr. 2020;53:102109. doi: 10.1016/j.ajp.2020.102109.

147. Ray S, Bhattacharya T, Das DK. Knowledge of auxiliary nurse midwives on immunization safety: a cross-sectional study in a sub-division of Purba Bardhaman district, West Bengal. Healthline. 2019;10(1):49-54.

148. Ria BA, Baso YS, Ramadhany S, Idris I, Bahar B, Jibril. The student role improvement in the learning process through the E-learning model in associate degree of midwifery, megarezky university. Eur J Mol Clin Med. 2020;7(7):4914-31.

149. Risnah, Amiruddin R, Mustamin, Irwan M, Mukhtar M, Nurhidayah, Gani NF, Hadrayani E, Muthahharah, Yustilawati E. Increasing midwifery capability on maternal health service. Gac Sanit. 2021;35:S519-S23. doi: 10.1016/j.gaceta.2021.10.084.

150. Rodrigo ESS, Wimalaratne SRU, Marasinghe RB, Edirippulige S. A pilot health information management system for public health midwives serving in a remote area of Sri Lanka. J Telemed Telecare. 2012;18(3):159-63. doi: 10.1258/jtt.2012.SFT109.

151. Romo A, Morgan M, Christmas A, Jacob A, Frank H, Dyer J, Spindler H, Walker D. Improving quality of obstetric and neonatal care through midwife mentoring and simulation training in Bihar, India: Mentor knowledge assessments. Ann Glob Health. 2016;82(3):353-4.

152. Rosa EM, Rahmi A, Hidayat A. Innovation in midwifery practices: Measurement of postpartum blood loss. Enferm Clin. 2020;30(S6):264-7. doi: 10.1016/j.enfcli.2020.06.060.

153. Rosmaria, Indrayani, Anggraini Y, Lestari BC, Kartikawati SL, Astuti LP, Ritha A. How Indonesian midwives assess blood loss during labor? Pak J Med Health Sci. 2019;13(4):1124-7.

154. Rukmini, Mikrajab MA, Pratiwi dNL. Gambaran sikap bidan terhadap kebijakan jampersal di kabupaten sampang. Bul Penelit Sist Kesehat. 2013;16(4):445-56.

155. Samidah I, Murwati, Handayani TS. Knowledge, perception, motivation and expectations of nursing and midwifery students on exit exam in Bengkulu City, Indonesia. Indian J Forensic Med Toxicol. 2021;15(3):3502-9.

156. Saragih E. The effect of semantic mapping technique on technical vocabulary mastery for midwifery students. Studies in English Language and Education. 2019;6(2):333-42. doi: 10.24815/siele.v6i2.14786.

157. Sardillah, Ahmad M, Manapa ES, Massi MN, Hadju V, Usman AN. Increased Leopold palpation knowledge and skills after learning using the preceptorship method. Enferm Clin. 2020;30(S2):606-8. doi: 10.1016/j.enfcli.2019.07.171.

158. Sari DJ, Lestari P, Mulawardhana P. The performance of midwives in early detection of cervical cancer using visual inspection test with acetic acid. Majalah Obstetri Ginekologi. 2022;30(2):52-7. doi: 10.20473/mog.V30I22022.52-57.

159. Sethi R, Tholandi M, Amelia D, Pedrana A, Ahmed S. Assessment of knowledge of evidence-based maternal and newborn care practices among midwives and nurses in six provinces in Indonesia. Int J Gynaecol Obstet. 2019;144(S1):51-8. doi: 10.1002/ijgo.12735.

160. Sharma B, Johansson E, Prakasamma M, Mavalankar D, Christensson K. Midwifery scope of practice among staff nurses: a grounded theory study in Gujarat, India. Midwifery. 2013;29(6):628-36. doi: 10.1016/j.midw.2012.05.008

161. Sharma B, Hildingsson I, Johansson E, Prakasamma M, Ramani KV, Christensson K. Do the pre-service education programmes for midwives in India prepare confident ‘registered midwives’? A survey from India. Glob Health Action. 2015;8(1):29553. doi: 10.3402/gha.v8.29553.

162. Sharma B, Hildingsson I, Johansson E, Christensson K. Self-assessed confidence of students on selected midwifery skills: Comparing diploma and bachelors programmes in one province of India. Midwifery. 2018;67:12-7. doi: 10.1016/j.midw.2018.08.015.

163. Stellata AG, Rinawan FR, Winarno GNA, Susanti AI, Purnama WG. Exploration of Telemidwifery: An Initiation of Application Menu in Indonesia. Int J Environ Res Public Health 2022;19(17):10713. doi: 10.3390/ijerph191710713.

164. Sugino M, Hapsari ED, Madyaningrum E, Haryant F, Warsini S, Takada S, Matsuo H. Issues raised by nurses and midwives in a post-disaster Bantul community.Disaster Prev Manag. 2014;23(4):420-36. doi: 10.1108/DPM-05-2013-0086.

165. Sujana IM, Hanafi N, Wilian S, Syahrial E, Fitriana E. Negotiating conflicts of needs in designing teaching english for midwifery students. Int J Lang Educ. 2019;3(2):20-6. doi: 10.26858/ijole.v3i2.10657.

166. Sumiaty S, Ali MN, Muhammad H, Hafid F. Roles of Midwives and Indonesian Midwives Association in Reducing Risk Factors for Stunting in Indonesia. Open Access Maced J Medical Sci. 2021;9(G):8-13. doi: 10.3889/oamjms.2021.6400.

167. Surianto S T, Widayanti DE. Legal protection for independent midwife for using ultrasonography in wonosobo regency. Indian J Public Health Res Dev. 2018;9(6):379-84. doi: 10.5958/0976-5506.2018.00582.X.

168. Susanti AI, Ali M, Hernawan AH, Rinawan FR, Purnama WG, Puspitasari IW, Stellata AG. Midwifery Continuity of Care in Indonesia: Initiation of Mobile Health Development Integrating Midwives’ Competency and Service Needs. Int J Environ Res Public Health. 2022;19(21):13893. doi: 10.3390/ijerph192113893.

169. Susianti N, Hayati TV. Hubungan karakteristik, sikap dan persepsi bidan terhadap penggunaan partograf di kabupaten tanjung jabung timur. Bul Penelit Sist Kesehat. 2017;20(4):133-40.

170. Thacker N, Choudhury P, Gargano LM, Weiss PS, Pazol K, Vashishtha VM, Bahl S, Jafari HS, Kumar A, Arora M, Venczel L, Orenstein WA, Omer SB, Hughes JM. Attitudes and Practices of Auxiliary Nurse Midwives and Accredited Social Health Activists in Uttar Pradesh and Bihar Regarding Polio Immunization in India. J Trop Pediatr. 2013;59(4):266-73. doi: 10.1093/tropej/fmt008.

171. Than KK, Mohamed Y, Oliver V, Myint T, La T, Beeson JG, Luchters S. Prevention of postpartum haemorrhage by community-based auxiliary midwives in hard-to-reach areas of Myanmar: a qualitative inquiry into acceptability and feasibility of task shifting. BMC Pregnancy Childbirth. 2017;17:146. doi: 10.1186/s12884-017-1324-6.

172. Than KK, Morgan A, Pham MD, Beeson JG, Luchters S. Determinants of knowledge of critical danger signs, safe childbirth and immediate newborn care practices among auxiliary midwives: A cross sectional survey in Myanmar. BMJ Open. 2017;7:e017180. doi: 10.1136/ bmjopen-2017-017180.

173. Than KK, Tin KN, La T, Thant KS, Myint T, Beeson JG, Luchters S, Morgan A. The potential of task shifting selected maternal interventions to auxiliary midwives in Myanmar: a mixed-method study. BMC Public Health. 2018;18(1):99. doi: 10.1186/s12889-017-5020-2.

174. Than KK, Luchters S, Tin KN, La T, Beeson J, Morgan A. The salt between the beans: a qualitative study of the role of auxiliary midwives in a hard-to-reach area of Myanmar. BMC Health Serv Res. 2019;19: 138. doi: 10.1186/s12913-019-3958-8.

175. Titaley CR, Wijayanti RU, Damayanti R, Setiawan AD, Dadun, Dachlia D, Siagian F, Suparno H, Saputri DAY, Harlan S, Wahyuningrum Y, Storey D. Increasing the uptake of long-acting and permanent methods of family planning: A qualitative study with village midwives in East Java and Nusa Tenggara Barat Provinces, Indonesia. Midwifery. 2017;53(2017):55-62. doi: 10.1016/j.midw.2017.07.014.

176. Ulfa Y, Igarashi Y, Takahata K, Shishido E, Horiuchi S. Effectiveness of team-based learning on postpartum hemorrhage in midwifery students in Indonesia: A quasi-experimental study. Nurse Educ Today. 2021;105: 105015. doi: 10.1016/j.nedt.2021.105015.

177. Ulfa YI, Y.; Takahata, K.; Horiuchi, S. Effects of team-based learning about postpartum haemorrhage on learning outcomes and experience of midwifery students in Indonesia: A pilot study. Nurs Open. 2021;8(1):241-50. doi: 10.1002/nop2.623.

178. Van Der Wal R, Hatem M, Lynn ZKK, Zarowsky C. Transitioning into democracy: What contextual barriers and facilitators do auxiliary midwives perceive in myanmar's first point-of-care Mhealth project? Ann Glob Health. 2016;82(3):505.

179. Vedam S, Titoria R, Niles P, Stoll K, Kumar V, Baswal D, Mayra K, Kaur I, Hardtman P. Advancing quality and safety of perinatal services in India: opportunities for effective midwifery integration. Health Policy Plan. 2022;37(8):1042-63. doi: 10.1093/heapol/czac032.

180. Vohra K, Ramaswamy G, Yadav K, Jaiswal A, Gupta S, Khanam A. Feasibility of Real-Time Monitoring for Anemia Using Mobile Application Linked With Point-of-Care Testing Device. Cureus. 2022;14(11):e32006. doi: 10.7759/cureus.32006.

181. Wahidah N, Sulaeman ES, Budihastuti URS. Determinants of midwife performance in lactation management in Surakarta and Karanganyar, Central Java. J Health Policy Manag. 2018;3(1):26-33. doi: 10.26911/thejhpm.2018.03.01.04.

182. Wahyuni TS, Purba J, Batubara A. The role of medical-legal ethics in midwifery practice- a study conducted in Pematangsiantar city of North Sumatra-Indonesia. IIOAB J. 2017;8(S3):10-2.

183. Wangmo S, Suphanchaimat R, Htun WMM, Aung TT, Khitdee C, Patcharanarumol W, Htoon PT, Tangcharoensathien V. Auxiliary midwives in hard to reach rural areas of Myanmar: filling MCH gaps. BMC Public Health. 2016;16(1):914/. doi: 10.1186/s12889-016-3584-x.

184. Warriner I. Midwives, nurses and doctors performing MVA in Vietnam and South Africa and early medical abortion in Nepal: evidence from randomized, controlled trials. Int J Gyneacol Obstet; 2015;131(S5):E32-E33. doi: 10.1016/S0020-7292(15)30002-3.

185. Emily H. Weaver EF, Bruce J. Fried, Duncan Thomas, Stephanie B. Wheeler, and John E. Paul. Effect of village midwife program on contraceptive prevalence and method choice in Indonesia. Perspect Sex Reprod Health. 2013;44(4):389-409. doi: 10.1111/j.1728-4465.2013.00366.x.

186. Widyawati W, Jans S, Utomo S, van Dillen J, Janssen ALML. A qualitative study on barriers in the prevention of anaemia during pregnancy in public health centres: perceptions of Indonesian nurse-midwives. BMC Pregnancy Childbirth. 2015;15:47. doi: 10.1186/s12884-015-0478-3.

187. Wild KJ, Gomes L, Fernandes A, de Araujo G, Madeira I, da Conceicao Matos L, McDonald S, Taft A. Responding to violence against women: A qualitative study with midwives in Timor-Leste. Women Birth. 2019;32(4):e459-e466. doi: 10.1016/j.wombi.2018.10.008.

188. Wrammert J, Sapkota S, Baral K, KC A, Målqvist M, Larsson M. Teamwork among midwives during neonatal resuscitation at a maternity hospital in Nepal. Women Birth. 2017;30(3):262-9. doi: 10.1016/j.wombi.2017.02.002.

189. Wyatt S, Ostbye T, De Silva V, Long Q. Antenatal depression in Sri Lanka: a qualitative study of public health midwives’ views and practices. Reprod Health. 2022;19:23. doi: 10.1186/s12978-022-01330-z.

190. Yadav R, Jelly P, Sharma SK, Sharma R. Views of Nurses, Obstetricians, and Mothers Regarding Independent Nurse-Midwifery Practitioner: A Mixed-Method Study. J Neonatol. 2022;36(1):32-41. doi: 10.1177/09732179221081197.

191. Yanti Y, Claramita M, Emilia O, Hakimi M. Students' understanding of "Women-Centred Care Philosophy" in midwifery care through Continuity of Care (CoC) learning model: A quasi-experimental study. BMC Nurs. 2015;14(1). doi: 10.1186/s12912-015-0072-z.

192. Youngwanichsetha S, Kritcharoen S, Chunuan S, Kala S, Phumdoung S. Flipped Classroom Learning Experiences of Graduate Nursing Students in Advanced Pathophysiology and Pharmacology in Midwifery Course. Int J Nurs Educ. 2020;12(1):85-9. doi: 10.5958/0974-9357.2020.00018.5

193. Zaman RU, Khaled A, Sabur MA, Islam S, Ahmed S, Varghese J, Sherratt D, Witter S. Experiences of a new cadre of midwives in Bangladesh: findings from a mixed method study. Hum Res Health. 2020;18:73. doi: 10.1186/s12960-020-00505-8.

194. Zin T, Myint KH, Moe T, Lin NA, Jhonson P, Aung NN, Currie S. Rapid analysis of midwifery pre-service education in Myanmar. Int J Gynaecol Obstet; 2015;131(S5):E528 doi: 10.1016/S0020-7292(15)30004-7.

195. Zullianti N, Budiono DI, Adityawarman A, Lestari P. Aspects that shape the midwive' perception of vaginal birth after caesarean (VBAC). Indonesian Midwifery and Health Sciences Journal. 2022;6(2):123-37. doi: 10.20473/imhsj.v6i2.2022.123-137.
