# Supplementary material for: Strengthening midwifery in the South-East Asian region: A scoping review of midwifery-related research
Source: PLoS One. 2023 Dec 15;18(12):e0294294. doi: 10.1371/journal.pone.0294294 (PMC10723687; doi:10.1371/journal.pone.0294294)
Supplement: Attachment — Submitted filename: Response to Reviewers Oct 2023.docx [file pone.0294294.s004.docx]

**PONE-D-23-28039**

**Strengthening midwifery in the South-East Asian region: A scoping review of midwifery-related research**

**PLOS ONE**

Thank you for the opportunity to respond to these very helpful reviews. We really appreciate the timeliness of this review. We have addressed each comment in turn and have track changes in the manuscript.

*3. Please expand the acronym “WHO SEARO” (as indicated in your financial disclosure) so that it states the name of your funders in full.
This information should be included in your cover letter; we will change the online submission form on your behalf.*

Thank you. This is corrected.

*4. Thank you for stating the following financial disclosure: 
 "The WHO SEARO office commissioned the study and were part of the analysis and writing team."*

*Please state what role the funders took in the study.  If the funders had no role, please state: ""The funders had no role in study design, data collection and analysis, decision to publish, or preparation of the manuscript.""  If this statement is not correct you must amend it as needed.*

*Please include this amended Role of Funder statement in your cover letter; we will change the online submission form on your behalf.*

The WHO SEARO office were involved in the study design, interpretation and decision to publish. They are included as co-authors. We have added this in the Cover Letter and the manuscript.

*5. We note that you have indicated that data from this study are available upon request. PLOS only allows data to be available upon request if there are legal or ethical restrictions on sharing data publicly. For more information on unacceptable data access restrictions, please see* [*http://journals.plos.org/plosone/s/data-availability#loc-unacceptable-data-access-restrictions*](http://journals.plos.org/plosone/s/data-availability#loc-unacceptable-data-access-restrictions)*.*

*We will update your Data Availability statement on your behalf to reflect the information you provide.*

The data are all extracted from published papers into an Excel spreadsheet. This is available in Supplementary Table 2.

*6. Please ensure that you refer to Figure 3 in your text as, if accepted, production will need this reference to link the reader to the figure.*

This is now included.

7. Please include captions for your Supporting Information files at the end of your manuscript, and update any in-text citations to match accordingly. Please see our Supporting Information guidelines for more information: <http://journals.plos.org/plosone/s/supporting-information>.

This is now included.

**Additional Editor Comments:**

*Please be consistent with the use of active voice throughout the paper.*

 We have altered the text in a number of places to address this.

*I suggest you acknowledge the limitations associated with excluding sources reporting on midwifery programs without peer review. Many NGO and official institutions might have published this type of report.*

Thank you. We have added that.

*The first ten lines of the data charting section belong to the results as well as the reference to supplementary file 2.*

This is moved and a small section on data charting is now included.

*I suggest discussing the implications of your findings for how to approach midwifery programs in relation to following international standards or contextualising the programs in local conditions. There is an important movement for defining and imposing international standards which might not always align with the diversity that you identified in your review.*

Thank you. I have added this.

*It is also possible to discuss the significance of culturally safe approaches to midwifery practice. As you mentioned towards the end of page 10, understanding the local context and establishing a good relationship with communities is crucial. Even more, Indigenous groups in Australia and New Zealand have expressed their concerns about programs that disrupt local and indigenous communities, for example, with initiatives trying to replace traditional midwives.*

Thank you. I have added this.

*Reviewer #1: General comment
I want to start by congratulating the authors on this high-quality manuscript. This scoping review addresses an important issue and offers a broad perspective on midwifery care. While the review focuses on the South-East Asia Region, its findings are also applicable to other Regions. The methodology is exceptionally well-conducted and could serve as an example for other regions to follow. The manuscript is well-written and contains relevant messages that can contribute to the global advancement of midwifery. I eagerly anticipate the publication of this manuscript and will promote it as an exemplary resource for my master’s and PhD students.*

Thank you. We appreciate this positive comment and hope it will be a useful paper when published.

*Detailed comments
- Pdf pg 10: insert a space between outcomes and [1] (“Thus, empowering midwives by prioritising their education and training are crucial to achieving sustainable improvements in sexual, reproductive, maternal, neonatal and adolescent health outcomes[1].”)*

Corrected.

*- Pdf pg 11: personally, I would prefer to see the 5 phases better reflected in the titles of the paragraphs in the method section*

Thank you – this is a good suggestion. We have done this now.

*- Pdf pg 12: inconsistency in writing eleven / 11 countries*

Thanks for picking this up – I have used the numeral.

*- Pdf pg 12: inconsistency in writing South-East Asia region/ Region*

Thank you. I have corrected this.

*- Pdf pg 13: insert a space between possible and [11] (“Study quality was not appraised, as scoping reviews include a broad range of evidence sources and this is not usually possible[11].”)*

Thank you. I have corrected this.

*- Pdf pg 14: correction CPD = continuous professional development instead of continuous professional delivery (CPD)*

Thank you. I have corrected this.

*- Pdf pg 16: Is it possible that we miss a word in the following sentence? “It was clear that should be integrated into their communities, and communities be informed of the benefits of midwifery care.*

Thank you. I have corrected this.
